# Formulation, Optimization, and Comprehensive Characterization of Topical Essential Oil-Loaded Anti-Acne Microemulgels

**DOI:** 10.3390/gels11080612

**Published:** 2025-08-04

**Authors:** Adeola Tawakalitu Kola-Mustapha, Muhabat Adeola Raji, Yusra Abdulkarim Alzahrani, Noura Hatim Binsaeed, Doaa Rashed Adam, Ranim Abou Shameh, Noureldeen Mohammed Garaween, Ghada Garaween

**Affiliations:** 1 Department of Pharmaceutical Sciences, College of Pharmacy, Alfaisal University, Riyadh 11533, Saudi Arabiadoaar.adam@gmail.com (D.R.A.);; 2 Department of Microbiology and Immunology, College of Medicine, Alfaisal University, Riyadh 11533, Saudi Arabia; mraji@alfaisal.edu (M.A.R.); ggaraween@alfaisal.edu (G.G.)

**Keywords:** Acne Vulgaris, microemulgels, essential oils, topical

## Abstract

*Cutibacterium acnes* is linked to the prevalent inflammatory skin disorder known as Acne Vulgaris (AV). Some topical agents exhibit unfavorable side effects like dryness and skin inflammation, and antimicrobial resistance (AMR) poses an increasing risk to effective AV management. This study develops and characterizes stable topical essential oil (EO)-loaded microemulgels with in vitro validated antimicrobial activities against *C. acnes* ATCC 6919, providing a solid scientific basis for their effectiveness. These microemulgels, with their potential to serve as an alternative to AMR-prone synthetic agents, could revolutionize the field of acne treatment. The MICs of the EOs (citronella, tea tree, and lemongrass) against *C. acnes* were determined. EO-loaded microemulgels were developed using a blend of microemulsion and carbopol/hyaluronic acid gel in a ratio of 1:1 and characterized, and their stability was observed over three months. The MICs of citronella, tea tree, and lemongrass EOs were 0.08, 0.16, and 0.62% *v*/*v*, respectively. The microemulgels were whitish and smooth, with characteristic EO odors. They demonstrated pH values ranging between 4.81 ± 0.20 and 5.00 ± 0.03, good homogeneity, a spreadability of 9.79 ± 0.6 and 12.76 ± 0.8 cm^2^, a viscosity of 29,500 and 31,130 cP, and retained stability at 4, 25, and 40 °C. EO-loaded microemulgels were developed with the potential of *C. acnes* management. The formulation shows adequate potential for further pharmaceutical development towards translational adoption in acne management.

## 1. Introduction

Skin diseases are ranked as some of the most common disease states; they very often have low mortality rates but can significantly affect quality of life. As a result, the design and development of novel products to manage skin diseases is highly important, especially to reduce high treatment failure rates. Acne Vulgaris (AV) is a very common skin disease with an estimated prevalence of more than 200 million cases globally [1]. In the Kingdom of Saudi Arabia, a review of epidemiology suggests its incidence is increasing [2]. The potential risks associated with current treatments for AV, such as antibiotic resistance and skin irritation, highlight the need for safer and more effective alternatives. AV, a prevalent skin condition, manifests physically on the skin as blackheads, papules, comedones, and various types of pimples commonly found on the face, chest, and back [3]. AV is known to cause a significant amount of psychological distress. For example, in a study reported by Alanazi and colleagues, about 14.5% of the study population in the Arar region of the Kingdom of Saudi Arabia (mainly female secondary school students) had their quality of life affected due to AV [4]. AV is widely reported to be commonly triggered in adolescence by a few factors and is pathophysiologically characterized by the inflammation of the pilosebaceous unit. Problems associated with current chemotherapy include the risk of antibiotic resistance from systemic drugs and the associated side effects of skin peeling such as drying and inflammation as seen with popular anti-acne topical medications such as benzoyl peroxide and the risk of embryotoxicity and teratogenicity with isotretinoin.

*Cutibacterium acnes*, or *C. acnes* (formerly known as *Propionibacterium acnes*), is an aerotolerant anaerobic, Gram-positive bacterium frequently associated with sebum-producing regions of the human skin [5,6,7] and other sites such as the mouth, the gastrointestinal tract, and the eyes [6]. It is strongly associated with Acne Vulgaris, which is reported to trigger innate immune responses and cause increased sebum production and aberrant keratinocyte differentiation [8]. *C. acnes* plays a key role in the pathophysiology of AV and has thus encouraged the use of various antibiotics in the treatment of AV. Examples of such antibiotics include clindamycin and erythromycin [9]. *Cutibacterium acnes* plays a crucial role in the pathogenesis of Acne Vulgaris by producing enzymes such as lipases and proteases that contribute to the inflammatory response in acne lesions [10]. Studies have shown that inhibiting *C. acnes* with natural antimicrobials like essential oils can significantly reduce inflammation and improve acne symptoms. Essential oils like tea tree, citronella, and lemongrass have demonstrated effective antibacterial activity against *C. acnes* strains, offering a promising alternative to conventional antibiotics that are associated with resistance [11].

Evidence suggests the role of inflammation in various stages of AV at any stage of the development of acne lesions. It plays a role not only in the later stages when inflammatory papules, pustules, and nodules are present but also in the early stages when microcomedones and comedones appear [12]. There is thus a rationale for the development of drug formulations that target not only the bacterial cause of AV but also the inflammatory pathway.

There are currently different approaches to AV treatment, including topical and systemic agents. Topical agents like retinoic acid, salicylic acid, topical clindamycin, and benzoyl peroxide are used for their anti-acne properties. The systemic intervention involves using various antibiotics such as doxycycline, minocycline, and oral contraceptives. Due to the emerging concern for antibiotic resistance associated with systemic agents which seriously threatens AV management, topical agents are increasingly preferred [13]. However, some topical agents have been associated with undesirable side effects, such as skin irritation and dryness. This observation has led to the consideration of natural oils in acne management.

Essential oils have been shown to possess some antimicrobial property against a range of bacterial infections, with few to no studies reporting antimicrobial resistance, and there is a need to explore the use of medicinal plants or essential oils of known antibacterial properties as a therapeutic option against mild-to-moderate AV following the crucial drug design and development stages. Essential oils are preferred not only for their antimicrobial properties but also for their lipophilicity, which enhances penetration through the skin and increases contact time in the treatment of acne [14].

Tea tree oil, rich in terpenoids, is one example of a natural oil with antimicrobial properties; its antibacterial activity is strongly associated with disrupting the bacterial cell membrane’s structural integrity and function [15]. Clinical studies have demonstrated that tea tree oil is as effective as benzoyl peroxide in treating mild-to-moderate acne, with fewer side effects such as dryness and irritation [16]. Obtained from the plant *Melaleuca alternifolia*, tea tree oil has widely acclaimed benefits in the management of acne. Gas Chromatography–Mass Spectrometry (GC-MS) shows the presence of the following constituents: Limonene, γ-Terpinene, Citral-A, α-Terpinene, Gamma Terpinene, 4-terpineol, Cis-Sabinene hydrate, P-cyeme-8-ol, Aromadendrene, Bromo acetate, Cineol, P-cymene, α-Terpinolene, and α-pinene [17]. Terpinen-4-ol is the most significant component, largely responsible for the oil’s anti-acne properties due to its antimicrobial and anti-inflammatory effects, as has long been established. This long-standing research on tea tree oil should instill confidence in its efficacy. An early review by Carson et al. highlighted the antimicrobial properties of tea tree oil, with a focus on terpinen-4-ol, which is effective against a variety of skin pathogens, including those suspected to contribute to acne [15]. Similarly, an even older study compared the efficacy of tea tree oil (rich in terpinen-4-ol) to benzoyl peroxide, showing that tea tree oil is effective in reducing acne lesions with fewer side effects [18].

Lemongrass (*Cymbopogon flexuosus*) is a commonly used plant widely known for both its leaves and essential oil, the latter of which consists largely of phenolic compounds, flavonoids, alcohols, terpenes, and aldehydes. It is reported to have antibacterial, antifilarial, antifungal, and antimalarial properties [19]. The demonstrable antibacterial and anti-inflammatory activities of lemongrass rationalize its potential use in managing acne. On analysis via GC-MS, lemongrass oil is reported to contain citral (34–35%), neral (30–31%), β-Myrcene (11–12%), geraniol (5–6%), 1,3,4-trimethyl-3-cyclohexene-1-carboxaldehyde (2.2%), citronellol (1.34%), geranyl acetate (0.57%), and D-Limonene (0.03%), in the order of decreasing relative peak area [20]. Citral is the major component of lemongrass essential oil and is reported to be primarily responsible for its antimicrobial and anti-inflammatory activities [21].

Citronella (*Cymbopogon winterianus*) oil has also been shown, through in vitro studies, to exhibit the dose-dependent inhibition of *C. acnes* by disrupting the bacterial cell wall membrane [22]. Citronella contains bioactive constituents including Geraniol, Geranial, Eugenol, Geranyl acetate, Citronellol, Linalool, and trans-β-caryophyllene, among others [23]. Furthermore, GC-MS studies conducted by Anwar et al. reported the presence of D-Limonene, Citronellal, Citronellol, and Geraniol, among others [24]. Citronellal, Geraniol, and Citronellol appear to be the major components of citronella oil, mainly exploited for their pharmacological activities, and commercially as fragrances in perfumes. They may also be responsible for its antimicrobial and anti-inflammatory properties [25]. Not only are these compounds reported to have antibacterial property against *C. acnes*, but also studies have shown that they possess anti-inflammatory activity [26,27]. Tea tree oil is not left out, as studies have also reported its benefits in reducing inflammation [28]. The antibacterial and anti-inflammatory activities of these essential oils thus provide a good rationale for their use in the management of acne.

As much as these oils are of benefit in the management of acne, the choice of formulation also plays an important role in the treatment of AV. In recent years, gels based on synthetic or semi-synthetic and natural polymeric constituents have been formulated to treat various dermatology problems and cosmetology exploits [29]. However, their popularity as topical bases is hinged on their ease of application and minimized side effects (reduced serum concentration of drugs) when deployed. Emulsified gels (microemulgels) are a unique form of formulation made up of emulsion droplets incorporated into a gel matrix or an aggregation of such emulsion droplets. The microemulgel delivery system ensures better skin permeation, the sustained release of active compounds, and enhanced therapeutic efficacy, particularly in treating skin infections like acne [30].

In this study, a topical or transdermal route that facilitates the local delivery of the active principle is preferred over systemic delivery due to its non-invasiveness, avoidance of hepatic metabolism, and improved patient compliance. The importance of this research is inextricably linked to the third sustainable development goal on enabling good health and wellbeing not only by the removal of acne but also by the removal of the associated psychological stress.

## 2. Results and Discussion

### 2.1. Physicochemical Characteristics of Essential Oils

The physicochemical characteristics of essential oils are presented in Table 1.

### 2.2. Solubility

**Figure 1:**
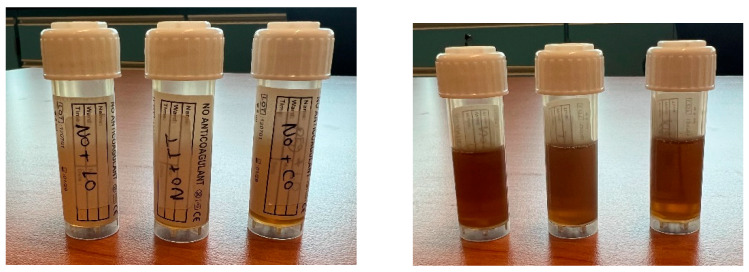
Solubilization of 1 mL of (tea tree/lemongrass/citronella) essential oil in 3 mL of neem oil. Clear solutions of the three essential oils (LO—lemongrass oil; TT—Tea Tree; CO—Cymbopogon oil) in neem oil were obtained, without any agglomeration or cloudiness observed.

**Figure 2:**
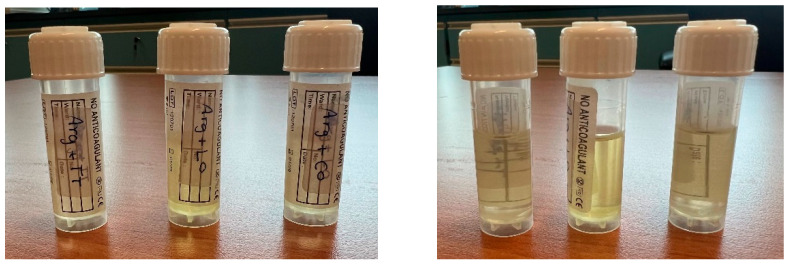
Solubilization of 1 mL of (tea tree/lemongrass/citronella) essential oil in 3 mL of argan oil. Clear solutions of the three essential oils (LO—lemongrass oil; TT—Tea Tree; CO—Cymbopogon oil) in argan oil were obtained, without any agglomeration or cloudiness observed.

An essential component of the emulsion formulation is oil, which can both increase the amount of lipophilic drug transportation and solubilize a noticeable amount of the lipophilic drug. The tea tree, citronella, and lemongrass essential oils (active components) required to prepare the emulsion component of the emulgel were dissolved using the argan oil. As a result, for an oil phase intended to improve emulsion, argan oil was chosen over neem oil (which showed partial solubility), as shown in Figure 1 and Figure 2.

### 2.3. Microbiology Pre-Formulation

**Figure 3:**
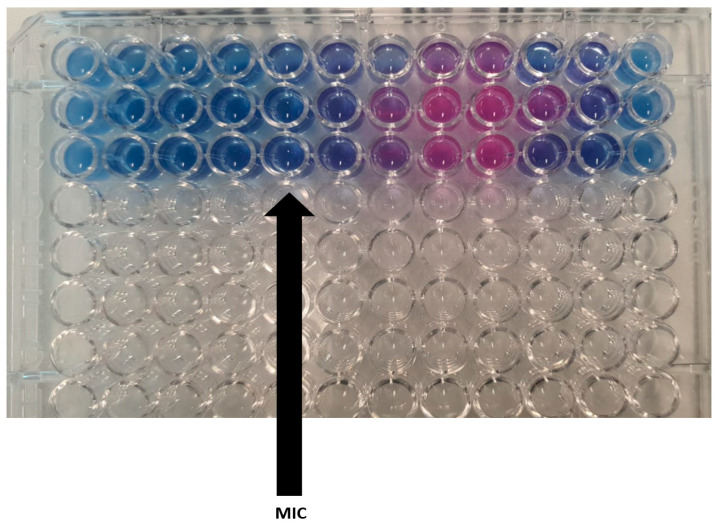
Microtiter plate showing the MIC for lemongrass oil in 10% Tween 80 (MIC = minimum inhibitory concentration).

MIC values were consistently the lowest with tea tree oil in triplicate MIC studies, compared to the other EOs, while citronella recorded the lowest MBC (Table 2). Our findings contrast with those of Bunrathep et al. In their study, lemongrass demonstrated comparatively higher inhibitory effects against *C. acnes* [31,32]. The microtiter plate demonstrating the triplicate findings of lemongrass oil MIC is shown in Figure 3.

### 2.4. Formulation and Optimization

Transcutol was favored as the co-surfactant due to its additional role as a permeation enhancer and PEG-40 as the surfactant. In addition to lowering interfacial tension, surfactants also provide a flexible film that easily stretches around droplets to facilitate the dispersion process. Co-surfactants give the interfacial film the flexibility it needs to spontaneously occupy the peculiar curvatures and structural conformations required to shape the emulsion over a wide composition range [33]. Deionized water was utilized as the aqueous phase.

#### HLB Values of Different Smix Ratios

The HLB values of various surfactant/co-surfactant (Smix) ratios (0:1, 1:1, 1:2, 1:3, 1:4, 2:1, 3:1, 4:1, and 1:0) were determined. It was observed that the HLB values increased as the concentration of the PEG-40 surfactant increased. In contrast, HLB values observably decreased with a decrease in the concentration of the co-surfactant, transcutol, as shown in Table 3. The Smix ratio of 2:1 was chosen with an HLB value of 11.45, which is close to the HLB value of argan oil (11 ± 1) [34,35].

**Figure 4:**
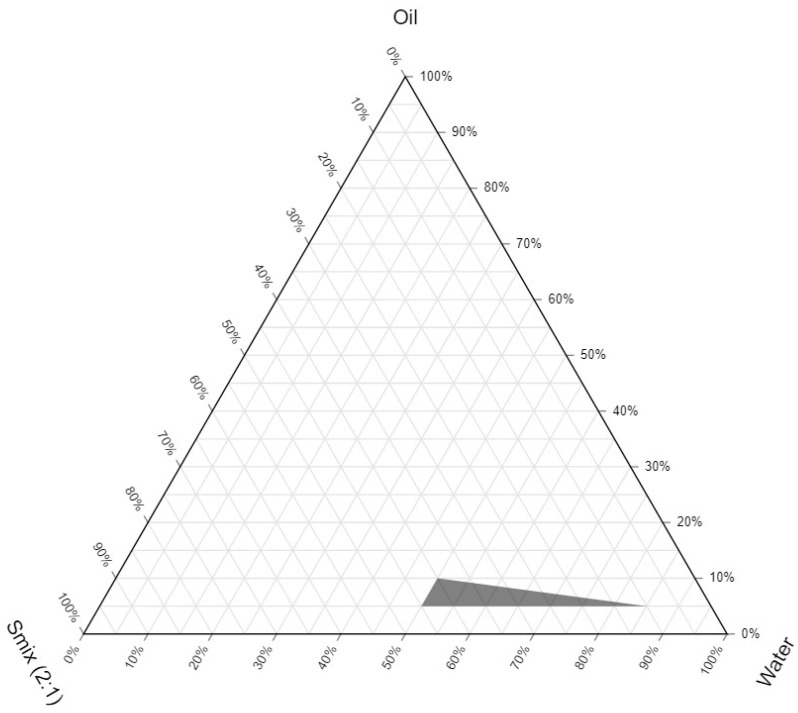
The pseudo-ternary phase diagram for an Smix ratio of 2:1. The shaded area represents the o/w emulsion region.

Optimization of the emulsion was achieved using the pseudo-ternary diagram (Figure 4). The pseudo-ternary phase diagram was produced using the aqueous titration method. The amount of aqueous phase added was mixed to produce a water concentration in the range of 5–95% of the total volume at a 5% interval. The pseudo-ternary phase diagrams were developed while using argan oil as the oily (i.e., dispersed) phase, combined with the Smix ratio (PEG-40 as a surfactant and transcutol as a co-surfactant) and deionized water as the aqueous (i.e., dispersion) phase. In the phase diagrams, only the oil-in-water (o/w) emulsion region is shown. The region of stable o/w emulsion is represented by the shadowed area in the built pseudo-ternary phase (Figure 4). Each corner of the phase diagram represents a 100% concentration of the corresponding constituent.

### 2.5. Stability of Microemulsions

#### 2.5.1. Kinetic Stability Findings

Instability in the form of creaming was visually observed in AE1, AE3, and AE4 freshly prepared microemulsions after subjecting them to the sequential centrifugation programs indicated in Table 4 at 15 min per speed. Only the AE2 microemulsion remained stable after the centrifugation processes at the three different speed levels (Table 4).

#### 2.5.2. Thermodynamic Stability

The emulsions were subjected to multiple thermodynamic studies (i.e., heating–cooling cycle, centrifugation, and freeze–thaw cycle) for 48 h in each cycle. The observations for thermodynamic stability studies are given in Table 5. Instability—in the form of either phase separation, creaming, or cracking, was visually assessed in the selected emulsions. The formulations which did not pass the thermodynamic tests (i.e., AE1, AE3, and AE4) were dropped, and the thermodynamically stable emulsion was put forward for the incorporation of the essential oils.

### 2.6. Physicochemical Characteristics of Microemulsions

The physicochemical properties of the microemulsions incorporated with the essential oils including droplet size, refractive index, conductivity, and viscosity are presented in Table 6.

### 2.7. Microscopy of Microemulsions

**Figure 5:**
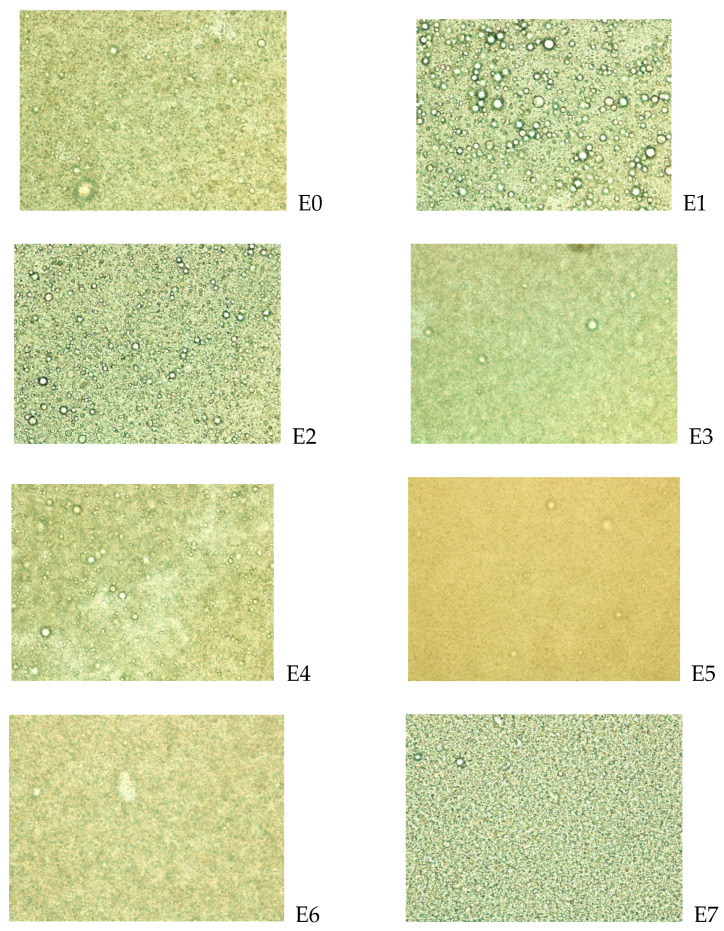
Microscopy of microemulsions E0–E7.

Microscopic pictures of various microemulsions are presented in Figure 5.

### 2.8. Physicochemical Properties of Microemulgels

In Table 7, the physicochemical properties of eight unloaded and loaded microemulgels are presented.

### 2.9. FTIR

**Figure 6:**
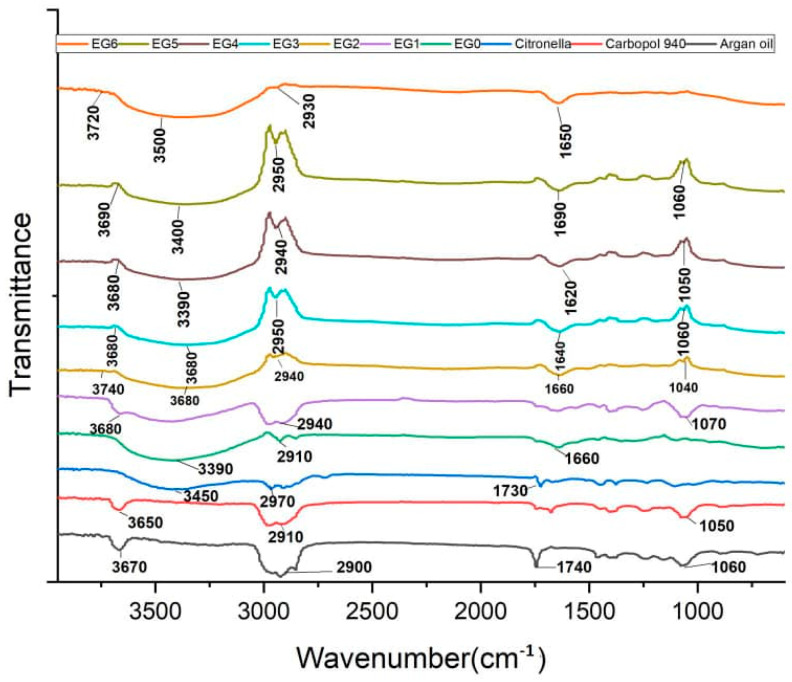
FTIR spectra of citronella oil, argan oil, carbopol 940, and EG0 to EG7 emulgel formulations.

The FTIR spectra of the samples are presented in Figure 6.

The peaks observed in the FTIR spectra of the respective formulations were annotated as illustrated (Figure 6) and assigned as presented in Table 8.

### 2.10. DSC

**Figure 7:**
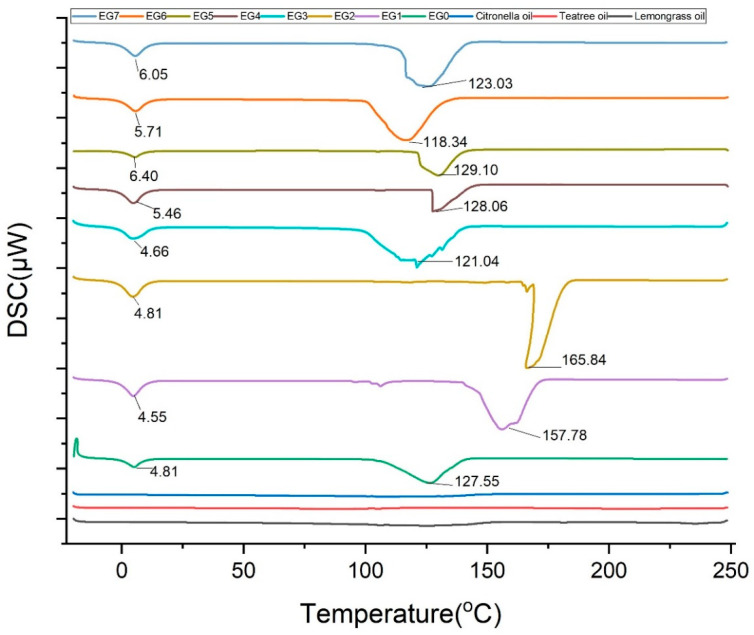
DSC thermograms of citronella oil, tea tree oil, lemongrass oil, and EG0 to EG7 emulgel formulations.

The DSC thermograms of the samples are presented in Figure 7.

### 2.11. Stability Testing of the Formulations

In Table 9, Table 10 and Table 11, the results of the observations for the stability of optimized microemulgel EG7 at 8, 25, 40 °C + 75% relative humidity (RH) are presented.

### 2.12. Antimicrobial Activities of the Microemulgel Formulations

Table 12 shows the measured inhibition zones of the various preparations in 1, 5, and 10% Tween 80 dilutions

### 2.13. Discussion

Physicochemical evaluations of the selected essential oils of *Cymbopogon winterianus* (citronella), *Melaleuca alternifolia* (tea tree), and *Cymbopogon flexuosus* (lemongrass) showed that they were clear, aromatic liquids with good solubility in PEG-40, transcutol^®^, and argan oil in a 1:3 ratio. The density values ranged from 0.892 g/mL (citronella) to 0.984 g/mL (lemongrass), consistent with values reported by Dhifi et al. [36], confirming their typical volatile oil profiles. Importantly, the consistency in refractive index, as observed, suggests that the formulated microemulsions are optically isotropic, implying compositional uniformity and the successful formation of stable microemulsion systems with consistent phase behaviors across the various formulations. Similarly, we observed high conductivity values that further confirm that the emulsions formed are indeed oil-in-water emulsions. The lemony aroma of citronella and lemongrass oils is attributed to their high citral content, as also noted in the work of Pavela and Benelli [37]. The relatively higher density of *C. flexuosus* may enhance its retention and controlled release in hydrogel systems, making it a strong candidate for oral antifungal formulations. These results collectively support the suitability of the selected essential oils for developing stable microemulgels for Acne Vulgaris because of these characteristics as well as their antibacterial and anti-inflammatory actions [38,39].

The solubility screening revealed that argan oil effectively dissolved all three essential oils—tea tree, citronella, and lemongrass—making it a suitable choice for the oil phase in the microemulgel formulations. Compared to neem oil, which demonstrated only partial solubility, argan oil offered a clear, homogenous dispersion, aligning with previous reports highlighting its favorable miscibility and emulsifying potential for essential oils [40]. The selection of an appropriate oil phase not only impacts the physical stability of the emulgel but also influences drug release and skin permeation properties [36]. Therefore, argan oil’s superior solubilizing capability supports its use as a functional excipient in the formulation of essential oil-based emulgels intended for antifungal therapy.

The FTIR analysis identified several chemical groups (hydroxyl (O–H), carbonyl (C=O), and alkene (C=C) groups, among others), which are significant for the biological activities of the EOs and microemulgel formulations. The presence of strong, broad O–H stretching bands around 3400 cm^−1^ in both the base and essential oil-loaded emulgels reflects contributions from phenolic and alcoholic constituents, particularly from citronellol, geraniol, and terpinen-4-ol, which are known to be abundant in citronella, lemongrass, and tea tree oils, respectively. These compounds exhibit antimicrobial activity by disrupting bacterial membranes and denaturing proteins, thereby inhibiting the growth of *C. acnes*, implicated in acne pathogenesis. Carbonyl bands observed between 1730 and 1650 cm^−1^ in the essential oil-loaded emulgels (EG1–EG7) correspond to aldehydes and ketones such as citral (from citronella and lemongrass oils), which are also noted for their antimicrobial and anti-inflammatory properties. These groups can act via electrophilic interactions with microbial proteins or enzymes, contributing further to antibacterial action. The alkene (C=C) peaks between 1650 and 1620 cm^−1^ also reflect unsaturated terpenes and contribute indirectly to membrane permeabilization or oxidative stress mechanisms that impair microbial viability [41,42].

The FTIR spectra reveal overlapping and, in some instances, slightly shifted peaks—particularly in the hydroxyl and carbonyl regions. Such shifts, notably those in the O–H band (~3400 cm^−1^) and C=O band (~1730 cm^−1^), suggest intermolecular hydrogen bonding or van der Waals interactions between the essential oils and excipients such as carbopol, argan oil, or hyaluronic acid. These interactions are desirable, as they indicate physical compatibility and stable integration of oils into the polymeric emulgel matrix. For example, a slight red shift in the O–H stretch in EO-loaded formulations relative to EG0 supports hydrogen bonding between the phenolic OH groups of EOs and the carboxyl groups of carbopol or the hydroxyl groups in hyaluronic acid. This can enhance gel consistency and oil dispersion, which are critical for uniform drug delivery and topical efficacy.

Both the minimum inhibitory concentration (MIC) and minimum bactericidal concentration (MBC) results for the essential oils show varying levels of antimicrobial efficacy against *C. acnes* ATCC 6919. Based on the MIC value, tea tree oil demonstrated the highest inhibitory activity against the organism with the lowest MIC (0.016% *v*/*v*) but a high MBC (1.000% *v*/*v*), suggesting it is highly effective in inhibiting bacterial growth, but requires a higher concentration for complete bactericidal activity. These findings align with prior studies that have identified terpinen-4-ol—i.e., the active component in tea tree oil—as responsible for its potent antimicrobial properties [43]. Citronella and lemongrass oils also displayed demonstrable antimicrobial activity, with citronella showing an MIC of 0.078% *v/v* and an MBC of 0.125% *v*/*v*, and lemongrass with an MIC of 0.062% *v/v* and an MBC of 0.25% *v*/*v*. Lemongrass oil’s efficacy is supported by previous research attributing its antimicrobial activity to citral, a major constituent with strong bactericidal properties [44]. Citronella’s effectiveness at relatively low concentrations has similarly been reported in the literature, where its use in topical antimicrobial formulations has been explored [38].

Based on the post-formulation zone of inhibition values, the combination of tea tree oil and citronella oil (EG4) demonstrated the most inhibitory activity against the organism under laboratory conditions. This observation aligns with studies that have reported terpinene-4-ol, the active component in tea tree oil, as a potent antimicrobial compound [45]. However, formulations incorporating citronella oil alone (EG3) show no inhibitory activity against the *C. acnes* strain used in this study. A loss of antimicrobial activity during the formulation process may be a possible explanation for this observation. These results suggest that, while tea tree oil is highly effective at low concentrations, lemongrass and citronella also offer potent antimicrobial effects, making them suitable alternatives or complements in formulations targeting *C. acnes* ATCC 6919.

The formulation and optimization of emulsions using transcutol as a co-surfactant and PEG-40 as a surfactant yielded promising results, particularly in enhancing the stability and flexibility of the emulsion. The choice of transcutol was strategic, as it not only acts as a co-surfactant but also serves as a permeation enhancer, facilitating the dispersion process by lowering interfacial tension and providing a flexible film around droplets [46]. This also aligns with the previous literature reports that noted that co-surfactants are essential for stabilizing emulsions and accommodating the unique curvatures required for effective dispersion across varying compositions [47].

The hydrophilic–lipophilic balance (HLB) values of the different Smix ratios indicated a direct relationship between the concentration of PEG-40 and increasing HLB values. The selected ratio of 2:1, with an HLB value of 11.45, is optimal for forming a stable oil-in-water emulsion, as this value is close to the HLB of argan oil (around 11). These findings align with previous research that highlights the importance of matching HLB values to the oil phase for creating stable emulsions [48]. In comparison, formulations with higher PEG-40 concentrations (e.g., 4:1) displayed excessively high HLB values, which may lead to instability in emulsions due to excessive hydrophilicity.

The pseudo-ternary phase diagram demonstrated the stable oil-in-water emulsion region using an Smix ratio of 2:1. The presence of a defined o/w emulsion region in the diagram suggests a robust formulation, supporting the use of argan oil as the oily phase combined with the selected Smix ratio. The shaded area confirms that the chosen Smix ratio supports stable emulsions, aligning with findings from other researchers who have successfully employed similar techniques to visualize and optimize emulsion systems [49]. This approach supported the successful formulation of microemulsions with varying oil and surfactant concentrations, crucial for tailoring the system’s drug-loading and delivery properties.

The stability evaluation of microemulsions AE1 to AE4 revealed that only AE2 maintained both kinetic and thermodynamic stability under stress conditions such as centrifugation, heating–cooling, and freeze–thaw cycles. The other formulations (AE1, AE3, and AE4) exhibited instability in the form of creaming or phase separation, particularly at higher centrifugation speeds and during thermal stress. This outcome supports previous reports [50,51], which emphasized the vulnerability of emulsions to destabilization under physical and thermal stress. The superior stability of AE2 may be attributed to the optimized Smix ratio (PEG-40/transcutol, 2:1), which achieved an HLB value close to that of argan oil, ensuring better emulsification and droplet stability.

The physicochemical evaluation of the developed microemulsions revealed droplet sizes ranging from 4.48 ± 2.59 µm to 8.28 ± 4.72 µm, indicating efficient emulsification. E0, E1, and E2 had the smallest droplet sizes, which can enhance drug release and penetration due to an increased surface area—a finding consistent with the earlier reports by Shakeel et al. [52], who emphasized that smaller droplet sizes improve stability and bioavailability in topical microemulsions. It was also observed that some of the droplet sizes recorded (e.g., for E1 and E3) had standard deviation values that are relatively high. This can be a consequence of sub-optimal uniformity in the samples analyzed, attributable to isolated experimental errors in sampling or temperature fluctuation during analysis. Microscopic analysis of the microemulsions (E0–E7) showed uniformly dispersed, spherical droplets, indicating good emulsification and stability [52]. All formulations displayed a consistent refractive index (1.07–1.08), confirming their optical transparency and isotropic nature, which is typical of thermodynamically stable microemulsions [53]. Electrical conductivity values between 94.3 and 106 µS/cm further validated the formation of oil-in-water (o/w) systems, considering the established correlation of higher conductivity with a continuous aqueous phase [54]. Viscosity remained within a narrow range (22–25 cP), supporting the ease of spreadability without compromising the formulation’s stability. These findings demonstrate that the formulated microemulsions possess desirable physical characteristics for topical and mucosal applications, aligning with criteria established in previous microemulsion research [50].

The optimized microemulgel formulation (EG7), comprising 2% carbopol 940 and 1% hyaluronic acid, demonstrated favorable physicochemical properties, including appropriate pH (4.81–5.00), viscosity (29.5–31.13 cP), spreadability (up to 12.7 cm^2^), and extrudability (up to 166.7 g/cm^2^), making it suitable for topical application. The formulation maintained good homogeneity, smooth texture, and a compatible pH for skin use. The high conductivity values also confirmed the presence of a continuous aqueous phase, supporting the o/w nature of the microemulsion system [52,54]. The use of hyaluronic acid contributed to improved skin feel and hydration, consistent with its known dermatological benefits [55]. Increased carbopol concentrations also demonstrably enhanced the formulation’s viscosity and structural integrity, consistent with its pharmaceutical use as a gelling agent and an emulgel stabilizer [56].

The FTIR analysis of the microemulgel formulations confirmed the chemical compatibility of active ingredients (citronella, tea tree, lemongrass oils) with excipients (carbopol 940, hyaluronic acid, and argan oil). Characteristic functional groups, including O–H, C–H, C=O, and C–O stretching vibrations, remained unaltered in both the individual components and the final formulations (EG0–EG7), indicating no significant interactions or degradation. The consistent presence of key peaks across spectra suggests that the essential oils were physically, not chemically, incorporated into the emulgel matrix.

The DSC analysis of microemulgel formulations also revealed multiple thermal transitions, indicating complex but stable phase behavior. Most formulations exhibited a low-temperature peak around 5–6 °C, corresponding to the initial lipid rearrangements, and a high-temperature peak (105–167 °C) linked to more stable crystalline transitions. The high enthalpy values observed, particularly in EG1 and EG2, suggest strong molecular interactions and thermal stability. These findings are consistent with previous reports [50,52], who demonstrated that lipid-based systems with structured matrices show distinct melting behaviors indicative of stability. Citronella oil also showed dual thermal peaks, affirming its compositional complexity. The absence of peak shifts or degradation in the loaded formulations indicates no thermal incompatibility, supporting their robustness under storage and usage conditions.

The physical stability study of the optimized microemulgel (EG7) demonstrated that the formulation remained stable at 8 ± 5 °C and 25 ± 5 °C for 90 days, showing no changes in color, homogeneity, or pH, and exhibiting a gradual increase in viscosity, indicating a stable gel matrix. In contrast, storage at 40 ± 5 °C resulted in physical degradation after week 8, including color change, phase separation, and a significant decrease in viscosity, suggesting that high temperatures adversely affect the structural integrity of the formulation. These findings are consistent with previous reports indicating that emulgel systems are more stable at cooler temperatures and susceptible to destabilization under heat stress [50,52]. However, despite the observed physical changes, pH remained stable across all conditions, indicating the chemical compatibility of the formulation components. Overall, EG7 showed excellent stability under ambient and refrigerated conditions, supporting its suitability for topical application with recommended cool storage.

## 3. Conclusions

In this study, we present a patient-centered alternative anti-acne formulation that could revolutionize the management of acne, allowing for phyto-pharmaceutical-based topical management of the skin disorder, replacing resistance-prone antibiotics like clindamycin with associated side effects. Our current findings demonstrate that the combination of citronella, tea tree, and lemongrass essential oils loaded into a microemulgel formulation offer a promising natural treatment for Acne Vulgaris. These essential oils exhibit strong antibacterial activity against *C. acnes*, as well as anti-inflammatory and sebum-regulating properties. The microemulgel system can enhance the delivery and efficacy of these oils, potentially offering a safer and more effective alternative to conventional acne treatments. The translation of this formulation to patients would allow for the better management of young adults, the sub-population in whom the disease is more prevalent. However, further studies characterizing active principle release profile and kinetics, in vivo biocompatibility, and animal models, among others, are required to further ascertain the translational potential of the developed formulation.

## 4. Materials and Methods

### 4.1. Materials

Argan oil, tea tree oil, citronella, and lemongrass oil were purchased from (Piping rock, Ronkonkoma, NY, USA). Hydrogenated castor oil 40, transcutol (Sigma Aldrich, Gillingham, UK), Carbomer 940 (Myoc), and triethanolamine were purchased from Sigma Aldrich (St. Louis, MI, USA), and deionized water was procured from Qualkem Ltd., Crewe, UK. Sterile cation-adjusted Mueller–Hinton agar (CAMH) plates were purchased from Saudi Prepared Media Limited (SPML). All other chemicals and reagents used in the study were procured from Merck and were of analytical (AR)-grade.

### 4.2. Pre-Formulation

#### 4.2.1. Physicochemical Characterization of Essential Oils

Based on the methods described in a previous study by Zekri et al., the physicochemical characteristics of the three essential oils, namely *C. witerianus* (citronella), *M. alternifolia* (tea tree) and *C. flexuosus* (lemongrass)—were evaluated. The solubility of the essential oils was determined using PEG-40, transcutol, and argan oil in a ratio of 1:3. The density was determined using a pycnometer [40].

#### 4.2.2. Microbiology Pre-Formulation Studies

- Essential oils

Essential oils (EOs) of lemongrass (*Cymbopogon flexuosus*), tea tree oil (*Melaleuca alternifolia*), and citronella oil (*Cymbopogon winterianus*) were obtained commercially from Piping rock (Ronkonkoma, New York, NY, USA). The physicochemical properties of the EOs were determined.

- Strain of Cutibacterium acnes

The control strain of *Cutibacterium acnes* (*C. acnes* ATCC 6919) used in the assay was stored in skimmed milk at −80 °C until use. The isolate was subcultured on trypticase soy agar and incubated in an anaerobic jar (Oxoid, Basingstoke, UK) with the addition of two packs of AnaeroPack-Anaero (Mitsubishi Gas Chemical, Tokyo, Japan) for improving assay performance.

- Preparation of essential oil working solutions

The neat EOs were diluted to a working concentration of 1/10 *v*/*v* by adding 100 µL of the EO to 1 mL of 1%, 5%, and 10% Tween 80. These dilutions of Tween 80 were tested separately pre-analysis and were found to have no growth-inhibiting effect on the *C. acnes* strain used.

- Serial dilution of solubilized EOs

The minimum inhibitory concentration (MIC) was determined visually by following the antibiotic broth microdilution protocol, which was described by Wiegand et al., with minor modifications [32]. The various EOs of lemongrass (*Cymbopogon flexuosus*), tea tree oil (*Melaleuca alternifolia*), and citronella oil (*Cymbopogon winterianus*) were solubilized in non-growth-inhibiting dilutions of 1%, 5%, and 10% of Tween 80.

Briefly, 50 µL of Mueller–Hinton broth (MHB) was pipetted into wells A2 to A10 of a 96-well microtiter plate with a lid (Corning, NY, USA). A 100 µL aliquot of the solubilized EO in Tween 80 was pipetted into well A1 of the 96-well microtiter plate and serially diluted by pipetting 50 µL from well A1 to well A2, and 50 µL from well A2 was pipetted into well A3. Serial dilution was continued until well A10. A 50 µL volume was discarded after the final dilution in well A10. Wells A11 and A12 were the growth (no essential oil) and sterility control (no bacterial inoculum) wells, respectively. Well A11 contained 100 µL of *C. acnes* in MHB, while well A12 contained 100 µL of the EO in Tween 80.

The bacterial inoculum added to the microtiter wells was prepared using a fresh culture of *C. acnes* ATCC 6919 on trypticase soy agar. First, a 0.5 MacFarland turbidity standard was prepared. This was further standardized to 1 × 10^5^ cfu/mL.

Ten microliters of well A11 and A12 contents were subcultured for growth and sterility on Mueller–Hinton agar. The microtiter plate was covered with an adhesive film and a lid and incubated under anaerobic conditions for 72 h. The assay was performed in triplicate.

After 72 h of incubation, the MIC was visually determined by adding 100 µL of 10% Alamar blue to each well, and the microtiter plate was incubated for one hour. A change in the reagent’s color from blue to pink indicated growth. The first well to show a color change represented the minimum inhibitory concentration (MIC) for the specific EO. MBC was determined by subculturing from this well to the last well with visual growth inhibition. The concentration showing no growth on subculture was recorded as the MBC. Subcultures were made in duplicates.

### 4.3. Formulation Optimization

#### 4.3.1. Selection of Components

Phase solubility studies were conducted to determine the appropriate oil to be used for the preparation of essential oil emulsion. Three milliliters of selected solubilizers/oils—i.e., PEG-40, transcutol, neem oil, and argan oil—were taken in small vials (5.0 mL capacity), and one milliliter of either tea tree, citronella, or lemongrass essential oils was added in the solubilizers and set aside in a biological shaker (Daihan Labtech Co., Ltd., Gyeonggi-do, Republic of Korea) for 72 h at a fixed temperature (25 ± 1.0 °C) to achieve equilibrium. The samples were detached from the shaker and centrifuged at 3000 rpm for 15 min.

#### 4.3.2. Determination of HLB Values

The measurement of the hydrophilic–lipophilic balance (HLB) was used to calculate the quantity of surfactant needed for the oil to remain in a solution. The HLB value indicates the ideal ratio of surfactant-to-oil required to form a stable emulsion, regardless of whether it is an oil-in-water (o/w) or water-in-oil (w/o) emulsion. The HLB value required for a stable o/w emulsion (8–18) was calculated using Equation (1):

(1) $$HLB=\frac{\left(Wa\times HLBa)+(Wb\times HLBb\right)}{Wa+Wb}$$

where Wa represents the amount (i.e., weight) of the first surfactant (i.e., PEG-40), Wb shows amount (i.e., weight) of the second surfactant (i.e., transcutol), and HLBa and HLBb represent the hydrophilic–lipophilic balance of surfactants a and b, respectively.

#### 4.3.3. Pseudo-Ternary Phase Diagram Construction

A pseudo-ternary phase diagram was composed to assess and analyze the phase behaviors of the various formulation components. A mixture with a volume ratio of 2:1 surfactant (PEG-40) and co-surfactant (transcutol) (i.e., Smix) was prepared, and 100 mL stock solutions from each fraction and various mixtures of oil (argan oil) and Smix were made in unique modified volume ratios ranging from 1:9 to 9:1 (i.e., 1:9, 1:8, 1:7, 1:6, 1:5, 1:3.5, 1:3, 1:2, 1:1, 2:8, 3:7, 4:6, 5:5, 6:4, 7:3, 8:2, and 9:1). The combined solution of argan oil and Smix was then titrated against deionized water. A visual inspection was conducted after every 5% addition of aqueous phase to the oil and Smix mixture, and the results of the inspection were recorded. Finally, the data were recorded and plotted on a phase diagram, where one axis characterized the aqueous phase, while the other represented the oil phase, and the third represented Smix. Based on the succeeding points, distinct formulations were identified and selected from the phase diagram produced for the Smix ratios. The oily phase was chosen at an appropriate concentration to easily dissolve the essential oils used as the active ingredients. The argan oil concentration from the phase diagram was selected as a numeral of five (i.e., 5, 10, 15, and 20%), and different concentrations (i.e., 10 to 30%) of surfactant Smix PEG-40/transcutol (2:1) were taken for microemulsion preparation as presented in Table 13.

### 4.4. Stability of Emulsion

#### 4.4.1. Kinetic Stability

The kinetic stability of AE1 to AE4 microemulsions were determined using the centrifugation method [57]. The samples were subjected to centrifugation at 1000, 2000, and 3000 rpm for 15 min at room temperature (25 °C). The formation of the microemulsion and phase separation both before and after the centrifugation cycle were recorded to measure the formulation’s kinetic stability.

#### 4.4.2. Thermodynamic Stability Studies

The thermodynamic stability of the microemulsion was determined using a cooling–heating cycle [58]. Samples were held at 4 °C (fridge) for 48 h and then at 48 °C (oven) for 48 h. The emulsion was then examined for any changes such as phase separation. Furthermore, emulsions were subjected to thermodynamic stability stress tests in the form of heating–cooling cycle, centrifugation, and freeze–thaw cycle. Heating–cooling cycles ran between 45 °C (oven) and room temperature (25 ± 2 °C), with a storage time of 24 h at each temperature (six cycles each), followed by centrifugation (5000 rpm for 30 min), and finally, freeze–thaw cycles were completed by storing the samples below −20 °C in a deep freezer (Vest frost, Hyderabad, India) and thawing them at room temperature (25 ± 2 °C) for 24 h (six cycles each).

### 4.5. Preparation of Microemulsion Loaded with Essential Oil

The preparation of essential oil-loaded microemulsion TAE involved blending 0.08/0.16/0.63% *w*/*w* of the citronella/tea tree oil/lemongrass oil with argan oil (based on kinetic and thermodynamic stability findings) to make a total of 10% oil component using a Stuart SA8 vortex mixer (Bibby Scientific, UK) and a 15% Smix ratio. The respective compositions of E0–E7 based on the incorporation of the three essential oils are as follows: E0: no essential oil (control); E1: citronella; E2: tea tree; E3: lemongrass; E4: citronella and tea tree; E5: tea tree and lemongrass; E6: citronella and lemongrass; and E7: citronella, tea tree, and lemongrass. Deionized water was then incorporated until a stable emulsion was achieved.

### 4.6. Physicochemical Properties of Microemulsions

#### 4.6.1. Globule/Droplet Size

A drop of each of the microemulsion samples was placed on a slide and stained with Giemsa’s staining solution (Loba Chemie, Mumbai, India). OLYMPUS DP73 BX53 microscope incorporated with cellSens Entry software (Olympus Corporation, Center Valley, PA, USA) was used to view and record the images of the microemulsions at ×100 magnification, and the droplet sizes were measured. Every measurement recorded was the average of three determinations, presented as mean ± SD.

#### 4.6.2. Refractive Index

After calibration, the refractive index of the microemulsion was identified using a digital handheld refractometer (Shen Tech, Shenzhen, China). Every measurement recorded was the average of three determinations, presented as mean ± SD.

#### 4.6.3. Microemulsions’ Conductivity

Conductivity meter (NanJing Tech, Nanjing, China) at 25 ± 1 °C was used to calculate electrical conductivity of the microemulsion and microemulgel formulations. Every measurement recorded was the average of three determinations, presented as mean ± SD.

#### 4.6.4. Microemulsions’ Viscosity

The microemulsion formulations’ viscosities were measured at 25 ± 2 °C and 50 rpm using the DV3T Brookfield viscometer (Ametek Brookfield, Middleboro, MA, USA) spindle F 96. Every measurement recorded was the average of three determinations, presented as mean ± SD.

### 4.7. Preparation of Emulgel

The following major steps were conducted during emulgel preparation [59].

#### 4.7.1. Preparation of the Gel

Two gelling agents were used, i.e., carbopol 940 and hyaluronic acid. To create the gel base, varying concentrations (i.e., 1, 1.5, and 2%) of carbopol 940 were added to deionized water and continuously stirred at room temperature (25 °C) until the gelling agent was fully dissolved. A 1% hyaluronic acid gel was also prepared with deionized water. The use of various concentrations of carbopol 940 was explored to assess the influence of concentration on both the viscosity and spreadability of the resulting emulgel. The different concentrations, i.e., 1, 1.5 and 2%, of carbopol were combined with 1% hyaluronic acid as shown in Table 14.

#### 4.7.2. Incorporation of the Prepared Gel into Already Prepared Microemulsion

A prepared TAE microemulsion was added in a dropwise fashion into the selected gel combination (2% carbopol/1% hyaluronic acid) coded G7 (optimized), as shown in Table 14, in a ratio of 1:1. It was then stirred for approximately 20 min at room temperature (25 °C) to convert it into an emulgel. Finally, the pH of the emulgel was adjusted to be close to the pH of the skin (5.5) with triethanolamine.

#### 4.7.3. Optimization of the Emulgel Formulation

The formulation of the emulgel was optimized based on three properties: viscosity, spreadability, and concentration of the carbopol 940 gelling agent. To optimize the formulation, seven different preparations were formulated with fixed concentrations of 2% carbopol 940 and 1% hyaluronic acid, G7 (Table 15).

### 4.8. Physicochemical Properties of the Microemulgels

#### 4.8.1. Physical Appearance

Visual observations were made based on the color, homogeneity, consistency, and phase separation of each microemulgel formulation.

#### 4.8.2. pH Determination

Using a pH meter (Hanna, Lincolnshire, UK), the pH of microemulgel formulations was measured by immersing the electrode tip in 1% microemulgel diluted in water. Every measurement recorded was the average of three determinations, presented as mean ± SD.

#### 4.8.3. Microemulgels’ Conductivity

Conductivity meter (NanJing Tech, Nanjing, China) at 25 ± 1 °C was used to calculate electrical conductivity of the microemulgel formulations. Every measurement recorded was the average of three determinations, presented as mean ± SD.

#### 4.8.4. Microemulgels’ Viscosity

The viscosity of the microemulgel formulations was measured at 25 ± 2 °C and 15 rpm using the DV3T Brookfield viscometer (Ametek Brookfield, Middleborough, MA, USA) spindle F (96). Every measurement recorded was the average of three determinations, presented as mean ± SD.

#### 4.8.5. Spreadability

The spreadability of the microemulgel was determined by modifying the parallel plate method, where 1 g of gel was placed between two horizontal plates (10 × 10 cm). To achieve a homogenous layer of formulation and release any air trapped between the two slides, a 100 g weight was placed on the upper plate, and the emulgel was allowed to spread for 1 min. Following this, the diameter of the spread was measured and the area of spread determined.

#### 4.8.6. Extrudability

The area of the extruded microemulgel was measured after 10 g of the gel formulations was put into collapsible tubes and squeezed out by applying a 1 kg load weight to the tube’s folded end, measuring the emulgel ribbon’s length, and applying the following formula: $Extrudability=\frac{Weight\;of\;Load\;(in\;grams)}{Area\;of\;ribbon\;extruded\;(in\;cm\;squared)}$; thus, the extrudability of the microemulgel was determined [59]. Every measurement recorded was the average of three determinations, presented as mean ± SD.

#### 4.8.7. FTIR Analysis

FTIR analysis was conducted to detect any incorporation incompatibilities between the formulation’s active ingredients and the excipients. FTIR analysis was conducted on carbopol 940, citronella, tea tree, lemongrass essential oil, and argan oil in both loaded and unloaded microemulgel formulations.

Approximately 10 mg of each formulation was placed onto the diamond surface plate of the Nicolet iS10 FTIR Spectrometer with a diamond Attenuated Total Reflectance (ATR) unit (Thermo Scientific, Waltham, MA, USA). The air of the laboratory environment was used as a blank. Sufficient pressure (i.e., 100–120 units) was applied to the gel samples for close contact compression. The spectrum for all samples—including the controls—was recorded within the wave range of 4000–400 cm^−2^, at an average of 32 scans, and a resolution of 0.4 cm^−1^. All measurements taken were performed in six replicates.

#### 4.8.8. Differential Scanning Calorimetry (DSC)

Hitachi DSC 7020 machine with an Intracooler Cooling Accessory (Hitachi High-Tech Sciences, Tokyo, Japan) was used to evaluate the thermal behavior of the formulated microemulgels. Indium and zinc standards were used to calibrate the temperature and heat flow of the instrument. The samples ranging from 5 to 8 mg were heated in hermetically sealed aluminum pans under nitrogen flow (50 mL/min), using a scanning rate of 10 °C/min from −20 to 250 °C. An empty aluminum pan was used as a reference. All measurements were performed an average of four times and expressed as mean ± SD.

#### 4.8.9. Physical Stability

Physical stability studies were conducted for all formulations at 4 ± 2 °C, 25 ± 2 °C, and 40 ± 2 °C. All the samples were transferred to tubes, tightly sealed, and stored at the above varying concentrations. The samples were evaluated for physical stability, changes in color, pH, homogeneity/separation, consistency, centrifugation, and viscosity at predetermined time intervals for 90 days.

### 4.9. Antimicrobial Activities of Microemulgels

A six-millimeter (6 mm) agar cork borer was used to punch holes into the medium. The punched holes were sealed using 10 µL of freshly prepared sterile cation-adjusted Mueller–Hinton agar (CAMH). The test emulgels (100 µL) were introduced into the wells after the surface had been inoculated with 0.5 MacFarland turbidity standard of the fresh growth of the bacteria. Neat emulgels without EOs were assayed separately. The inoculated plates were incubated at 37 °C for 72 h under anaerobic conditions. Zones of inhibitions were measured in millimeter using a ruler, and the mean ± SEM was determined and recorded.

### 4.10. Statistical Analysis

All the data were analyzed using the GraphPad Prism software, version 8.0.

## Figures and Tables

**Table 1:** Physicochemical analysis of the utilized essential oils (EOs).

Essential Oil	Color	Clarity	Form	Odor	Solubility	Density (g/mL)
*C. witerianus*	Light yellow	Clear	Liquid	Lemony	+	0.892
*M. alternifolia*	Colorless	Clear	Liquid	Pungent	+	0.898
*C. flexuosus*	Golden yellow	Clear	Liquid	Lemony	+	0.984

+: soluble.

**Table 2:** MIC for the various essential oils before incorporation in the emulgels.

Essential Oil	MIC (*v*/*v*%)	MBC (*v*/*v*%)
Citronella	0.078	0.125
Tea tree	0.016	1.000
Lemongrass	0.062	0.25

**Table 3:** Effect of Smix ratio on HLB value.

Smix Ratio (PEG-40/Transcutol)	HLB Value
0:1	4.20
1:1	9.60
1:2	7.76
1:3	6.90
1:4	6.36
2:1	11.45
3:1	12.30
4:1	12.84
1:0	15.00

**Table 4:** Kinetic stability of microemulsions formulated.

Microemulsion	1000 rpm	2000 rpm	3000 rpm
AE1	✓	✓	X
AE2	✓	✓	✓
AE3	X	X	X
AE4	✓	✓	X

Legend: ✓ = stable; X = unstable.

**Table 5:** Thermodynamic stability of formulated microemulsions.

Microemulsion	Heating-Cooling	Centrifugation	Freeze–Thaw
AE1	✓	X	X
AE2	✓	✓	✓
AE3	X	X	X
AE4	X	X	X

Legend: ✓ = stable; X = unstable. Heating–cooling cycle: 4–48 °C (48 h each, for 6 cycles). Centrifugation: 45 °C to room temperature (24 h each), then centrifugation at 5000 rpm for 30 min. Freeze–thaw: −20 °C to room temperature (24 h each, for 6 cycles).

**Table 6:** Physicochemical properties of the microemulsions.

Formulations	E0	E1	E2	E3	E4	E5	E6	E7
Droplet size + SD (μm)	4.48 ± 2.59	4.74 ± 3.58	4.69 ± 2.79	8.28 ± 4.72	5.44 ± 2.73	6.38 ± 3.63	6.60 ± 1.98	6.77 ± 2.76
Refractive index	1.08 ± 0.12	1.07 ± 0.05	1.08 ± 0.07	1.07 ± 0.11	1.08 ± 0.04	1.07 ± 0.09	1.07 ± 0.14	1.07 ± 0.18
Conductivity (µS/cm)	105.3 ± 1.2	102.7 ± 2.3	106 ± 2.0	97.3 ± 2.5	103.3 ± 3.1	102.0 ± 3.0	94.3 ± 3.1	105.3 ± 1.2
Viscosity (cP)	22.0 ± 0.92	23.0 ± 1.27	22.0 ± 1.32	23.0 ± 1.59	23.0 ± 1.22	22.0 ± 1.02	23.0 ± 0.82	25.0 ± 1.15

**Table 7:** Physicochemical properties of the microemulgels.

Formulations	EG0	EG1	EG2	EG3	EG4	EG5	EG6	EG7
Color	White	White	White	White	White	White	White	White
Texture	Smooth	Smooth	Smooth	Smooth	Smooth	Smooth	Smooth	Smooth
Odor	Nutty/earthy	Floral smell	Grassy smell	Citrus /pungent	Citrus /pungent	Grassy /floral	Lemon /pungent	Citrus /pungent
Homogeneity	Good	Good	Good	Good	Good	Good	Good	Good
pH	5.00 ± 0.03	4.94 ± 0.12	4.93 ± 0.08	4.95 ± 0.06	4.84 ± 0.14	4.94 ± 0.18	4.97 ± 0.10	4.81 ± 0.08
Conductivity (µS/cm)	843.3 ± 9.2	750 ± 32.9	749.7 ± 18.2	755.3 ± 30.1	774.3 ± 12.9	754.0 ± 0.0	759.3 ± 7.0	840.7 ± 5.9
Viscosity (cP)	29.50 ± 0.83	30.20 ± 0.66	30.80 ± 0.72	29.80 ± 0.42	30.90 ± 1.04	30.50 ± 0.55	30.70 ± 0.45	31.13 ± 1.25
Spreadability (cm^2^)	9.8 ± 0.6	11.8 ± 0.5	11.6 ± 0.8	12.1 ± 1.0	11.9 ± 1.1	12.4 ± 0.6	12.1 ± 0.2	12.7 ± 0.8
Extrudability (g/cm^2^)	133.3 ± 3.6	142.3 ± 1.8	141.3 ± 2.4	144.3 ± 1.6	148.3 ± 4.3	146.3 ± 2.8	155.3 ± 4.3	166.7 ± 2.3

**Table 8:** FTIR spectra peak assignments.

Wavenumber (cm^−1^)	Functional Groups/Bonds	Vibrational Modes	Likely Sources in Sample	Found In
3740–3650	O–H (free hydroxyl)	Stretching	Water, phenols in EOs	All analytes, except citronella and EG0
3600–3390	O–H (H-bonded hydroxyl, broad); N–H	Stretching	Carbopol, hyaluronic acid, triethanolamine, water	All analytes, except argan oil and carbopol
2950–2900	C–H (CH_3_, CH_2_)	Asymmetric/symmetric stretching	Argan oil, citronella, lemongrass, tea tree EOs, aliphatic EO chains, lipid chains, alkane groups	All analytes
1740–1730	C=O (ester/carboxylic acid)	Stretching	Argan oil esters, carbopol	Citronella and argan oils
1690–1620	C=O (aldehyde, ketone, amide I); C=C (alkene) or amide I	Stretching	Citral in citronella, hyaluronic acid backbone, EO ketones, terpenes (e.g., Limonene), hyaluronic acid	All EGs except EG2
1070–1040	C–O (alcohols, ethers)	Stretching	EO alcohols (geraniol, citronellol), carbopol ester	All analytes except EG0 and EG6
1060–1050	C–O–C (ether)/primary alcohol	Stretching	Argan oil, citronella, lemongrass EOs	EG5, EG4, EG3, carbopol, argan oil

**Table 9:** Stability of EG7 microemulgel at 8 ± 5 °C.

Week	Color	Homogeneity/Phase Separation	pH	Centrifugation	Viscosity (cP)
0	White	Homogenous	4.81 ± 0.08	Stable	31.13 ± 1.25
1	White	Homogenous	4.81 ± 0.08	Stable	31.90 ± 0.82
2	White	Homogenous	4.81 ± 0.08	Stable	32.22 ± 0.64
4	White	Homogenous	4.81 ± 0.08	Stable	33.93 ± 0.73
8	White	Homogenous	4.81 ± 0.08	Stable	34.02 ± 1.04
12	White	Homogenous	4.81 ± 0.08	Stable	36.42 ± 0.88

**Table 10:** Stability of EG7 microemulgel at 25 ± 5 °C.

Week	Color	Homogeneity/Phase Separation	pH	Centrifugation	Viscosity (cP)
0	White	Homogenous	4.81 ± 0.08	Stable	31.13 ± 1.25
1	White	Homogenous	4.81 ± 0.08	Stable	31.45 ± 0.82
2	White	Homogenous	4.81 ± 0.08	Stable	31.62 ± 0.64
4	White	Homogenous	4.81 ± 0.08	Stable	31.93 ± 0.73
8	White	Homogenous	4.81 ± 0.08	Stable	32.02 ± 1.04
12	White	Homogenous	4.81 ± 0.08	Stable	32.42 ± 0.88

**Table 11:** Stability of EG7 microemulgel at 40 ± 5 °C.

Week	Color	Homogeneity/Phase Separation	pH	Centrifugation	Viscosity (cP)
0	White	Homogenous	4.81 ± 0.08	Stable	31.13 ± 1.25
1	White	Homogenous	4.81 ± 0.08	Stable	30.96 ± 0.63
2	White	Homogenous	4.81 ± 0.08	Stable	29.42 ± 0.92
4	White	Homogenous	4.81 ± 0.08	Stable	27.58 ± 0.46
8	Off-white	Heterogenous	4.81 ± 0.08	Unstable	24.37 ± 0.78
12	Off-white	Heterogenous	4.81 ± 0.08	Unstable	20.22 ± 0.42

**Table 12:** Measured inhibition zones of the various preparations in 1, 5, and 10% Tween 80 dilutions.

Formulation	ZOI (mm) 1% Tween 80	ZOI (mm) 5% Tween 80	ZOI (mm) 10% Tween 80
EG0	6 ± 0	6 ± 0	6 ± 0
EG1	6 ± 0	6 ± 0	6 ± 0
EG2	15 ± 0	15 ± 0	15 ± 0
EG3	15 ± 0	20 ± 0	25 ± 0
EG4	20 ± 0	23 ± 0	25 ± 0
EG5	15 ± 0	23 ± 0	25 ± 0
EG6	20 ± 0	20 ± 0	20 ± 0
EG7	20 ± 0	20 ± 0	20 ± 0

Key: ZOI, zone of inhibition measured in mm; 6 mm = no inhibition zone; positive control = erythromycin 25 mm.

**Table 13:** Components selected for microemulsion based on the phase diagram.

Type	Oil (%)	Smix (%)	Water (%)
AE1	5	10	85
AE2	10	15	75
AE3	15	20	65
AE4	20	30	50

**Table 14:** Composition of the formulations of the gel.

Components	G1 (%)	G2	G3	G4	G5	G6	G7
Carbopol 940	1.0	1.5	2.0	-	1.0	1.5	2.0
Hyaluronic acid	-	-	-	1.0	1.0	1.0	1.0
Germall™ plus	0.1	0.1	0.1	0.1	0.1	0.1	0.1
Deionized water up to	100.0	100.0	100.0	100.0	100.0	100.0	100.0

**Table 15:** Composition of the formulations of the emulgel.

Components	Unloaded EG0 (%)	EG1 (%)	EG2 (%)	EG3 (%)	EG4 (%)	EG5 (%)	EG6 (%)	EG7 (%)
Citronella essential oil	-	0.08	-	-	0.08	-	0.08	0.08
Tea tree essential oil	-	-	0.16	-	0.16	0.16	-	0.16
Lemongrass essential oil	-	-	-	0.63	-	0.63	0.63	0.63
Argan oil	10.0	9.92	9.84	9.37	9.76	9.21	9.29	9.13
Carbopol 940	2.0	2.0	2.0	2.0	2.0	2.0	2.0	2.0
Hyaluronic acid	1.0	1.0	1.0	1.0	1.0	1.0	1.0	1.0
Germal plus	0.1	0.1	0.1	0.1	0.1	0.1	0.1	0.1
Triethanolamine	qs	qs	qs	qs	qs	qs	qs	qs
Deionized water up to	100.0	100.0	100.0	100.0	100.0	100.0	100.0	100.0

qs—quantity sufficient until the target pH is reached.

## Data Availability

The original contributions presented in this study are included in the article. Further inquiries can be directed to the corresponding author.
